# The immunoglobulin domain of *C. elegans* IGEG-2/EGF is required for its function

**DOI:** 10.17912/micropub.biology.001855

**Published:** 2025-10-23

**Authors:** Darlene Mendez, Marine Barsegyan, Cheryl Van Buskirk

**Affiliations:** 1 Biology, California State University, Northridge, Northridge, California, United States

## Abstract

Epidermal Growth Factor (EGF) family ligands mediate signaling events in development and physiology across species. These ligands are transmembrane proteins that undergo ectodomain shedding to release the soluble EGF domain, which mediates interaction with EGF receptors (EGFR). Some EGF ligand ectodomains also contain an immunoglobulin-like domain (IgD), and the function of this domain within Ig-EGFs varies. *C. elegans* IGEG-2 is an EGFR ligand of unknown function, identified and named for its Ig and EGF domains. The EGF domain appears to be functional, as widespread expression of IGEG-2 produces phenotypes associated with EGFR hyperactivation. Here we use these phenotypes to investigate the contribution of the IGEG-2 IgD, and we find it to be essential for IGEG-2 /EGF signaling. The mechanism underlying this strict IgD dependence is not known.

**Figure 1. The Ig domain of IGEG-2/EGF is required for EGF(OE) vulval induction and sleep:**
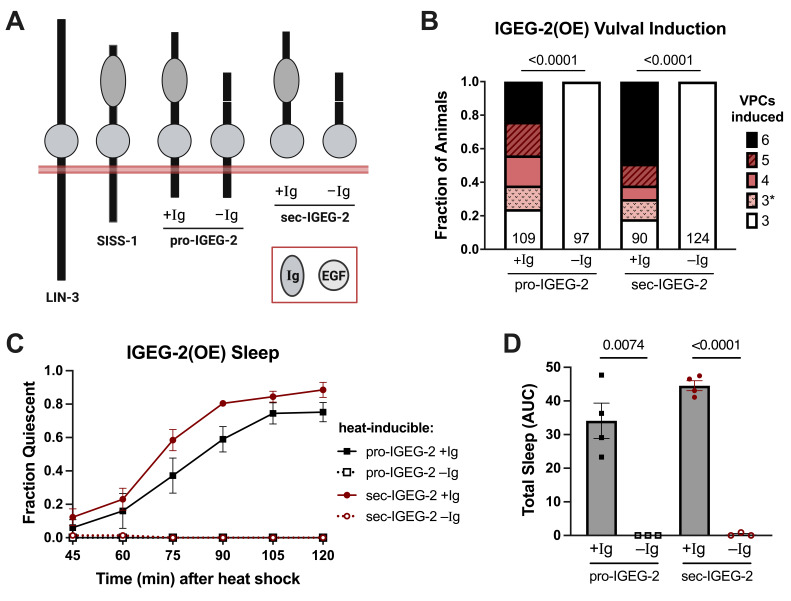
**(A)** Domain structures of the IGEG-2 constructs examined in this study, compared to the *C. elegans* EGFR ligands LIN-3 and SISS-1 . Ig = immunoglobulin-like domain. EGF = Epidermal Growth Factor-like domain. **(B)** Number of vulval precursor cells (VPCs) induced to adopt a vulval fate following overexpression (OE) of the IGEG-2 constructs depicted in panel A. Transgene expression was induced at the L2 larval stage and VPC induction was inferred by examination of vulval morphology in L4 larvae. 3* represents animals with an excess of primary-fated cells among P5.p-P7.p, with no additional VPCs induced. Numbers at the base of each bar indicate how many animals were examined. P values were determined by two-tailed Fisher's exact tests comparing 3 VPCs vs. hyperinduced (all other categories combined). **(C)** Time course of fraction of animals quiescent following transgene induction at the young adult stage. At least three trials of 25 animals per genotype were performed. **(D)** Total transgene-induced quiescence (area under curve, AUC) for data shown in panel C. P values were determined by unpaired t-tests with Welch's correction for unequal variances.

## Description

*Caenorhabditis elegans* possesses three known ligands of the Epidermal Growth Factor (EGF) family, encoded by *lin-3* (Hill & Sternberg 1992), *siss-1* (Hill et al. 2024), and *igeg-2* (Mailhot et al. 2025). LIN-3 mediates several signaling events including the induction of vulval cell fates, wherein LIN-3 released by the gonadal anchor cell activates LET-23 /EGFR within three of six multipotent vulval precursor cells, or VPCs (Moghal & Sternberg 2003). Transgenic overexpression of LIN-3 during mid-larval development leads to hyperinduction, with up to six VPCs adopting vulval fates (Katz et al. 1995). A second EGFR ligand, SISS-1 , appears to be released from a range of tissues in response to cellular damage to activate LET-23 /EGFR in sleep-promoting neurons, mediating stress-induced sleep, or SIS (Hill et al. 2024). Transgenic overexpression of SISS-1 (or LIN-3 ) at any stage produces a robust EGFR-dependent sleep bout characterized by a cessation of feeding and locomotion (Van Buskirk & Sternberg 2007; Hill et al. 2024). While LIN-3 and SISS-1 were identified by their mutant phenotypes, IGEG-2 was identified as a predicted transmembrane protein with immunoglobulin-like (Ig) and EGF domains in its ectodomain ( Fig. 1A ; Sternberg et al. 2024). All members of the EGF family undergo ectodomain shedding via processing within their juxtamembrane region (Higashiyama et al. 2008). *igeg-2* null mutants are superficially wild type and the function of IGEG-2 is not known. However, IGEG-2 overexpression (OE) can promote EGFR-dependent events including vulval cell fate induction and sleep (Mailhot et al. 2025), indicating that it is a functional EGFR ligand. Here we aim to use these overexpression phenotypes to perform a structure-function analysis of IGEG-2 .

EGF family ligands are characterized by the presence of an EGF domain, which participates in receptor binding via three loops formed by disulfide bonds among six highly conserved cysteines (Harris et al. 2003). Immunoglobulin-like domains (IgD) are β-sandwich structures common among cell surface proteins, and heterogeneous interactions between Ig domains regulate recognition, adhesion, and signaling in a variety of contexts (Halaby et al. 1999). Ig-like and EGF domains are found together in the ectodomains of *C. elegans* SISS-1 , *Drosophila* Vein, and certain vertebrate Neuregulins, but the impact of the Ig domain appears to vary (Jones & Van Buskirk 2025; Donaldson et al. 2004; Li & Loeb 2001). The IgD of SISS-1 is critically required for its sleep-promoting activity via an unknown mechanism (Jones & Van Buskirk 2025), and the IgD of human Neuregulin 1 confers enhanced and extended receptor activation (Li & Loeb 2001), likely via anchoring of the ligand to heparan sulfate proteoglycans within the extracellular matrix (Loeb & Fischbach 1995; Meier et al. 1998). By contrast, the IgD of Vein is not required for endogenous wing vein patterning, but expression of Ig-deleted Vein has toxic effects not seen following expression of native Vein, suggesting that the Ig domain may restrict inappropriate signaling (Donaldson et al. 2004).

To investigate the function of the IGEG-2 Ig domain, we expressed full-length and Ig-deleted variants of IGEG-2 ( Fig. 1A ) from a heat-inducible promoter in a wild-type background and assayed phenotypes associated with EGFR hyperactivation. We first examined vulva induction, normally mediated by LIN-3 released from the gonadal anchor cell during the L2 larval stage. This localized source of LIN-3 activates LET-23 /EGFR in the nearest three of six multipotent vulval precursor cells (VPCs) to initiate vulval organogenesis (Moghal & Sternberg 2003). Widespread expression of LIN-3 (Katz et al. 1995) or IGEG-2 (Mailhot et al. 2025) at the L2 stage from a heat-inducible promoter drives hyperinduction, with up to six VPC adopting vulval fates. We first examined the requirement for the Ig domain within the context of overexpression of a full-length *igeg-2* cDNA ( Fig. 1A, pro- IGEG-2 +Ig vs. –Ig) and we found the Ig domain to be essential for vulval hyperinduction ( Fig. 1B ). To test the possibility that the IgD functions to recruit a sheddase, we examined the IgD-dependence of a constitutively-secreted form of IGEG-2 ( Fig. 1A, sec- IGEG-2 +Ig vs. –Ig). We found that like pro- IGEG-2 , sec- IGEG-2 function is critically dependent on the IgD ( Fig. 1B ), pointing to a role other than sheddase recruitment. Interestingly, the fraction of hyperinduced vulvae produced by pro- IGEG-2 (OE) and sec- IGEG-2 (OE) is not significantly different (P=0.3831, Fisher's exact test of fraction hyperinduced), indicating that ectodomain shedding is not a limiting step in IGEG-2 signaling, at least not during overexpression.

We then examined whether the Ig domain is required for IGEG-2 (OE) signaling in the context of sleep-promoting neurons. Stress-induced sleep (SIS) is normally mediated by SISS-1 /EGF shedding from damaged cells by the stress-responsive protease ADM-4 , activating LET-23 /EGFR in the ALA and RIS neurons (Hill et al. 2024; Konietzka et al. 2020). Widespread expression of any *C. elegans* EGFR ligand, including IGEG-2 , induces a prolonged sleep bout (Van Buskirk & Sternberg 2007; Hill et al. 2024; Mailhot et al. 2025). We found the IGEG-2 IgD to be necessary for the sleep-promoting activity of both pro- IGEG-2 and sec- IGEG-2 ( Fig. 1C, D). Again, the phenotypes observed with overexpression of pro- IGEG-2 and sec- IGEG-2 are not distinct from each other (P=0.1046, unpaired t-test of area under the curve sleep data), indicating that ectodomain shedding is not a limiting step in IGEG-2 (OE) signaling.

Together our data indicate that the IGEG-2 Ig domain plays a crucial role in EGF signaling in multiple contexts. This IgD dependence is not attributable to a role in ectodomain shedding, at least not solely, leaving open possible functions in protein trafficking, ligand stability, and/or receptor interaction. Like IGEG-2 , the *C. elegans* Ig-EGF SISS-1 shows strict IgD dependence, as CRISPR-mediated deletion of the IgD abolishes endogenous stress-induced sleep (Jones & Van Buskirk 2025). These findings set *C. elegans* Ig-EGFs apart from those that have been characterized in other species, which retain activity without an IgD (Li & Loeb 2001; Donaldson et al. 2004). An understanding of the contribution of the IgD to IGEG-2 signaling will likely require the identification of the endogenous function of this EGFR ligand.

## Methods

**Vulval Induction:** Transgenic late L2 animals were transferred to 35 x 10 mm (5 mL) NGM plates seeded with OP50 *E. coli.* Plates were sealed with Parafilm and placed upright (lid up) in a 33°C water bath for 30 minutes to induce transgene expression. Animals were grown at 16˚C before and after heat shock and examined at the L4 stage by differential interference contrast (DIC) microscopy on a Zeiss Axio Imager A2. The number of VPCs induced was inferred by examination of invaginations of the ventral epithelium. For example, wild-type induction of 3 VPCs produces a characteristic invagination at the L4 stage containing 22 vulval cells, while maximal hyperinduction (6 VPCs) yields up to three more invaginations that together possess roughly another 22 vulval cells (Sternberg & Horvitz 1991). Experimenter was blind to genotype.

**Sleep Assays:** Transgenic young adult animals raised at 16˚C were transferred to 5 mL NGM plates seeded with OP50 *E. coli.* Plates were sealed with parafilm and placed in a 35 °C water bath for 20 minutes to induce transgene expression. After heat shock, parafilm was removed and plates were cooled to room temperature by placing them on frozen LabArmor beads for 1 min. Plates were moved to a stereomicroscope at room temperature for examination of transgene-dependent sleep. Animals with no pharyngeal pumping nor movement during a 3-second observation were scored as quiescent. Experimenter was blind to genotype.

**Transgene Construction:** The hs:pro- IGEG-2 transgene was constructed as previously described (Mailhot et al. 2025). The hs:pro- IGEG-2 (–Ig) construct was synthesized in a similar manner but with the Ig domain (residues 46-117, nucleotides 151-366 of the F48C5.1.1 transcript) deleted. Gene fragments corresponding to secreted IGEG-2 with and without the IgD were synthesized by Twist Biosciences as for pro- IGEG-2 but truncated 8 amino acids upstream of the predicted transmembrane domain, by replacing Val180 of the full-length ORF with a stop codon (TAA). Each fragment was flanked by KpnI and SacI sites and cloned into the vector pPD49.83 (Addgene plasmid #1448), placing each IGEG-2 variant under the control of the heat-inducible *hsp-16.41* promoter. The resulting plasmids (pCV50-53, included in the strain table) were injected into wild type N2 animals at 10 ng/ul by InVivo Biosystems, along with coinjection markers. At least three independent lines were examined for each, and representative lines were used in this study.

**Software:** The data was graphed and analyzed as described in the figure legend using GraphPad Prism software. The Figure 1A schematic was created in BioRender https://BioRender.com/oktmmft .

## Reagents

**Table:** 

**Strain name**	**Genotype**
CVB82	*csnEx7* [ *hsp-16.41* p::pro- IGEG-2 (pCV50), *col-12* p::DsRed]
CVB99	*csnEx14* [( *hsp-16.41* p::pro- IGEG-2 (–Ig) (pCV51), *myo-2* p::DsRED]
CVB104	*csnEx13* [ *hsp-16.41* p::sec- IGEG-2 (pCV52), *myo-2* p::GFP]
CVB101	*csnEx11* [ *hsp-16.41* p::sec- IGEG-2 (–Ig) (pCV53), *myo-2* p::GFP]

All strains are available from the Van Buskirk lab upon request.
